# Identifying barriers and benefits of patient safety event reporting toward user-centered design

**DOI:** 10.1186/2056-5917-1-7

**Published:** 2015-08-27

**Authors:** Yang Gong, Hsing-Yi Song, Xinshuo Wu, Lei Hua

**Affiliations:** School of Biomedical Informatics, University of Texas Health Science Center, 7000 Fannin St. Suite 165, Houston 77030, TX, USA

**Keywords:** Patient Safety, Reporting systems, Survey

## Abstract

**Background:**

To learn from errors, electronic patient safety event reporting systems (e-reporting systems) have been widely adopted to collect medical incidents from the frontline practitioners in US hospitals. However, two issues of underreporting and low-quality of reports pervade and thus the system effectiveness remains dubious.

**Methods:**

This study employing semi-structured interviews of health professionals in the Texas Medical Center investigated the perceived benefits and barriers from users who have used e-reporting systems.

**Results:**

As a result, the perceived benefits include the enhanced convenience in data processing and the assistant functions leading to patient safety enhancement. The perceived barriers to the acceptance and quality use of the system include the lack of instructions, lack of reporter-friendly classifications, lack of time, and lack of feedback The identified benefits and barriers help design a user-centered e-reporting system where learning and assistant features are discussed during the interviews.

**Conclusions:**

As a response, the learning and assistant features aiming at enhancing benefits and removing barriers of e-reporting systems should be included for facilitating the acceptance and effective use of the systems.

## Background

One-third of patients in the United States report experiencing medical, medication, or test errors [1]. It is estimated that 210,000-440,000 people suffer from preventable harms that contribute to their deaths each year [2]. In recent years, progress of enhancing patient safety has been made in raising awareness, developing reporting systems, and establishing national data collection standards. Multiple methods have been applied in addressing patient safety issues, including using administrative data, medical record abstraction, patient surveys, and spontaneous adverse event reports which allow a more robust understanding of what is improving and what is not [3].

One way that is most commonly used is the voluntary electronic patient safety reporting system (e-reporting), wherein event data are collected and aggregated in a properly structured format [4], for the detection of event patterns, discovery of underlying factors, and generation of actionable knowledge. As a repository of adverse events, the system enables patient safety researchers to categorize, trend, and analyze data for quality improvement and care enhancement in a timely and effective fashion [5].

Studies on the reporting behavior of healthcare professionals have shown that under-reporting is a major problem of e-reporting. Underreporting of adverse events is estimated to range from 50–96% annually [6,7]. For example, according to Hospital Survey on Patient Safety Culture [8]: 2012 User Comparative Database Report, though most events that could harm the patients (74% ± 6.91%) were thought to be reported, the events that have been corrected or had no potential harm were less reported (57% ± 8.83%, 59% ± 8.42%). Most respondents (55%) reported no events in the past 12 months. And there is a very limited increase (1%) in the number of people who have reported at least one event over the years [8]. Another issue impeding the wide adoption and utilization of e-reporting systems is the low quality of the reports [4,9]. Data quality is a multidimensional concept that depends on the context where data are produced, with accuracy and completeness being two of the most frequently mentioned aspects [10]. Unfortunately, the reports from e-reporting are suffering from incomplete and inaccurate data that are misleading and not usable for further analysis. Furthermore, it has been unclear what informatics approaches and human factors could help improve the completeness and accuracy of the reports.

Research on user interface design in e-reporting has received little attention. In order to enhance the use of e-reporting and improve data quality, we conducted this project to investigate barriers, benefits, and human factors toward better e-reporting through user-centered design. Meanwhile, we sought to evaluate the perception of our proposed user-centered features of encouraging quality reporting, promoting data completeness and accuracy, and facilitating learning from the reports.

## Methods

The research project was approved by the Committee for the Protection of Human Subjects (CPHS) of the University of Texas Health Science Center at Houston. Participants were healthcare professionals with the experience of using e-reporting. Participants were recruited by flyers and invitation emails where the purpose of the study and the inclusion criteria were explained. Inclusion criteria for this study were based on occupation and previous experience of using e-reporting which reflected the major groups of e-reporting users, including nurses, physicians, department managers, and technicians in hospitals [8]. Participant with minimum one time reporting experience was verified by questions in the study. Due to sensitive nature of voluntary reporting, no further demographic information was obtained from the participants.

### Questionnaire for participant screening

According to the Surveys on Patient Safety Culture (HSOPS) [8], we adapted the original survey questions into an online questionnaire aiming at screening interview candidate and training for the follow-up interview. The questionnaire included structured questions inquiring the participant’s role, the general evaluation of e-reporting, and preference of our proposed user-centered features, shown in Table 1. In addition, a group of assistant features, such as data extraction from institutional systems and dynamic content that only shows relevant information were also introduced. A five-point Likert scale was used in the questions where 1 indicated a minimal level of agreement of the statement and 5 indicated a maximal level. The questions in the online questionnaire are shown in Table 2. Short video clips were provided in the online questionnaire to facilitate understanding the designed features. The validity of the questionnaire was examined by two informatics faculty who are experienced in e-reporting, one being a physician, the other a nurse.

### Interviews

To explore benefits and barriers to e-reporting implementation and gain feedback on the proposed features, semi-structured interviews were designed and conducted in the study. Interviews were scheduled based on respondents’ availability by telephone or in person. The interviews were open-ended and guided by an interview script. The quality of the data collection process was enhanced by collecting and analyzing the data simultaneously. The interviewer transcribed verbatim immediately after each interview when time permits. The interviewer strongly felt that some semi-structured questions could be asked at a better point of time so that the interviewees may respond with more relevant details. The interview script included questions concerning the participants’ experience of e-reporting, perceived benefits and barriers, as well as the perceptions of our proposed features. Topics discussed in the interviews are listed in Table 3.

### Data collection

Data collection was conducted between April and July, 2013. Each interview took 15–30 minutes to complete and was compensated with a $25 gift card. All answers were recorded by field notes, then transcribed verbatim and stored in computers for analysis. The interview stopped until the thematic saturation was achieved.

### Data analysis

A thematic analytical approach was used in analyzing the interview responses [12]. The interviewer was appointed for the data analysis. Interview responses were reviewed for further understanding and coded with Nvivo version 10 during the initial line-by-line coding process. 103 codes were created and 296 quotes were cited from the interview transcript. The codes were further classified into themes in terms of perceived benefits and perceived barriers among the respondents. In the end, two themes were created for the benefits mentioned by the respondents and four themes were concluded to cover the reported barriers to e-reporting adoption. The perceptions of the proposed features were summarized additionally.

## Results

Forty-four interview candidates were recruited in the study, and 34 of them finished the online questionnaire. Of the ones who completed the screening and training questionnaire, 16 participants took part in the final interview section. In the end, nine nurses, three physicians, two department managers, and two clinical technicians were interviewed. A 15-page transcript was generated after the interviews.

In the interviews, the respondents’ perceived benefits of implementing e-reporting included enhanced convenience in data processing and the functions of e-reporting in improving patient safety. On the other hand, four leading barriers to e-reporting adoption were reported by users, including lack of instructions, lack of reporter-friendly classifications, lack of time, and lack of feedback. The main findings were summarized in Table 4.

### Perceived benefits of e-reporting

#### Convenience in data entry and sharing

When asked about the benefits of e-reporting, six respondents thought the convenient process of data entry was the primary advantage of e-reporting. Another aspect identified as the advantage of the system was the convenience of communication with risk managers and leaders provided by e-reporting, compared to paper-based systems, writing emails, and oral reporting. As one respondent explained:

“It (using e-reporting) may be better than verbally reporting to a manager or writing an email because the manager may not always have time to answer the call or meet you… Using the system is more convenient and comfortable. You can report the case whenever you have time.”

(Respondent 16)

#### Improvement in patient safety

E-reporting was thought to be helpful in improving patient safety from various perspectives. The content and analysis derived from the reports in e-reporting have helped the health care professionals to “find the problems”, “investigate the reasons of the incidents” and “give the right solutions”. (Respondent 6, 15, 8). On the other hand, eight respondents were not aware of the improvement in patient safety after the implementation of e-reporting or did not think it as a benefit of having e-reporting. As mentioned by one respondent:

“Since we have the reports we are still seeing the same number of events and seeing things happen again and again in the same location. There is just no improvement.”

(Respondent 6)

### Perceived barriers to the use of e-reporting

#### Lack of trainings of patient safety reporting and instructions in e-reporting

There was no training about patient safety reporting as mentioned by some respondents (N = 3). And the lack of training about the purpose and importance of error reporting could impact reporters’ decision to report. For example, one respondent considered the lack of education on the importance of e-reporting to be one reason behind under-reporting:

“It (patient safety reporting) also has something to do with their (reporters’) evaluation of the system and if they interpret more of it. Not everyone thinks it is important.”

(Respondent 8)

The lack of instructions on the analysis process of patient safety events also made the reporting process difficult for the reporters. One respondent had described a case that showed the difficult analysis needed in patient safety event reporting :

“A patient fell and you were not there… You need to think what word to use and how to describe the situation. But it can be very difficult since you were not there. Not to say to determine the cause of the fall. You need to be very careful and it is just very tough for me.”

(Respondent 14)

The insufficient tutorials and assistant features in e-reporting made it hard for users to report an event as well. Some reporters (N = 2) said they needed help from other colleagues in order to complete the report for the first time. One respondent mentioned the difficulty of accessing the system due to the lack of guide to log into the system:

“It is very hard to know where to go. There is a small icon on our website to log into the system. The most time-consuming part is finding where to go…”

(Respondent 11)

#### Lack of reporter-friendly classifications

The confusing classifications of analyzing the patient safety events have impacted the quantity and quality of the reports as pointed out by three respondents. Cases would be reported according to reporters’ personal judgment if the taxonomies used in e-reporting cannot be correctly perceived by the reporters. One respondent has mentioned the difficult process of classifying a case:

“The only thing (problem of e-reporting) is about the options in the system. Like there are three types of categories (of events) but sometimes the case does not match any of them. And I can just select ‘others’. The options are not enough.”

(Respondent 15)

#### Lack of time

Two prominent barriers to the use of e-reporting are the busyness and time in short of health professionals. They were common among nurses and physicians. As mentioned by a nurse and a physician:

“Nurses are very busy. We need to call the doctors, order X-rays, and take care of the patients. And when we finish all the tasks it is usually time for us to go home. There is just no time to write a report.”

(Respondent 14, a nurse)

“But people don’t use it (e-reporting). I think the primary barrier for physicians is still time. It is just because their days can be busy and unpredictable.”

(Respondent 8, a physician)

#### Lack of feedback

Ten of the 16 respondents (63%) had not received or did not remember if they had received the feedback about the cases they reported. Thirteen respondents (81%) had not received or did not remember if they had received feedback about the general patient safety conditions in their department or institutions. Respondents had expected feedback from e-reporting, but did not have access to the feedback after they submitted their reports. The content of feedback described by the respondents varied, including whether the manager has received the reports, who is in charge of reviewing the reports, whether the case is under review or investigation, the final analysis report (reason for the case, patient recovery, etc.), what action has been taken, prevention guidelines, department performance, frequent causes of events, and frequent types of events.

### Perceptions of the proposed learning features

The participants had positive attitudes toward our proposed user-centered learning features. Main perceptions about the four proposed user-centered learning features were shown in Table 5. These proposed user-centered features were considered to provide efficiency and guidance during the reporting process as well as facilitate learning from patient safety events. On the other hand, 15 participants (94%) have preferred at least one of the proposed assistant features.

#### The side panel that automatically summarizes the report

Eleven of the 16 respondents (69%) thought the side panel that automatically summarizes the report would be helpful during the reporting process. Most respondents provided positive evaluations of the side panel in terms of an enhanced control over the reporting process in e-reporting. The feature was considered to inform the reporters of the reporting progress and could be used to estimate the time needed to finish the report when using e-reporting. Since the reporter would be more aware of what was entered and what was left to be entered with the side panel, some respondents (N = 6) believed that the side panel would also be useful in guiding data entry. As mentioned by one respondent:

“The side panel is helpful. It helps you to recall the scenario and reminds you what is left to be entered.”

(Respondent 15)

Other respondents (N = 2) mentioned that the feature would be helpful in preventing errors because it provided the opportunity to check the major points of the report so that they can know what the final report was going to look like and whether any content was not entered or entered mistakenly.

“A side panel summarizes the report is beneficial because it allows you to check if the case is correctly written. The summary is good for checking accuracy.”

(Respondent 12)

#### The table showing similar cases

Thirteen of the 16 respondents (81%) thought the table showing similar cases would be useful. The similar cases were mainly perceived as examples to follow for new users of e-reporting and thus gave guidance and assistance to reporters who were not familiar with the system. By giving similar cases, the standard and format of the reports could be learned by the reporters, which could ensure the quality of data and save the time of thinking how to report a case. One respondent with English as a second language has explained the advantage of the table showing similar reports:

“The only thing bothers me is making the statement about the case. You need to be careful about the words you use… I think similar reports can be useful in giving some idea about how to report a case in the same situation. In my case, English is my second language. And it can be very helpful to me to get some idea of making the statement.”

(Respondent 14)

Another benefit of the table showing similar cases mentioned by the respondents was its function as a learning tool. Besides learning about how to report a case, the similar reports were thought to be immediate feedback to reporters with educational information. The analysis of similar cases and their prevention guidelines could help the reporters to react to the current case and prevent future errors. As mentioned by one respondent:

“You know here is the mistake and how to use this information to prevent what has been made. It can be an enforcement of what people have seen from their work.”

(Respondent 8)

#### The table showing the previous reports

Eleven of the 16 respondents (69%) thought the table showing the previous reports was important and helpful. These respondents were interested in the content presented in the table, which showed the reports previously submitted by the reporters along with the review status of the reports, the final analysis of the reports and the action being taken. The table showing the previous reports was closely linked to the lack of feedback among reporters. One respondent as a department manager talked about the lack of feedback when asked about why the feature would be helpful:

“I as a manager would like to see historical information and follow-up information. And that’s one of the problems that I think we have in our current system… There is no feedback. I would like to see any kind of feedback.”

(Respondent 6)

#### Statistics with graphics

Ten of the 16 respondents (63%) thought statistics with graphics would help. Statistics with graphics was expected to make it easier and faster to understand and share the analysis and conclusion of the reports. As mentioned by two respondents:

“… the chart and other graphics can help the person to quickly know the rates and types after they report the case. Graphics are better since they are more visual than text.”

(Respondent 4)

“It helps a lot if we can pick graphs that are already available instead of creating them by ourselves. It will save a lot of time. Currently a lot of management time is just entering data from multiple systems into Excel to form usable documentation to share.”

(Respondent 10)

## Discussion

### The perceived benefits, barriers, and possible facilitators

It has been shown that reporters’ attitude about the effectiveness of e-reporting is a major factor influencing the willingness to report [13]. Therefore, investigating the perceived benefits of e-reporting may be useful in exploring the motivators for future reporting. One important perceived benefit of e-reporting is the design that offers convenience in data entry and processing. The convenience was presented by the respondents in three aspects: guidance from structured questions, flexibility from open-ended questions and efficiency in data sharing. Guidance from structured questions may help retain consistency and standard of the reports. On the contrary, flexibility from unstructured questions provides richness and compensates what could be missed if only structured questions are used [14]. In addition, it is essential in healthcare and e-reporting design that the reporters can send their reports to multiple recipients immediately after completing the reports. Therefore, a system design that uses semi-structured questions with efficient data sharing function would help improve the perceived benefits of e-reporting among reporters.

Another perceived benefit is the effectiveness as a tool that promotes patient safety. Some respondents were aware of the functions that enhance patient safety and thus considered the system useful. However, the improvement in patient safety was not supported by the rest of respondents. Reporting systems may have multiple potential purposes, and one of the goals is the reduction of the errors through the analysis of the reports. Studies have shown that the lack of perceived usefulness would discourage reporting while having the perception that the system is useful promotes reporting [13,15,16]. If a reporting system provides functions and feedback to support the final improvement of patient safety, such as identifying errors, facilitating root cause analysis, providing educational information, etc. it may be thought to be significant and useful by the reporters, which helps encourage patient safety reporting.

Despite the benefits mentioned above, e-reporting contained large incomplete and inconsistent reports. Identifying the perceived barriers may be beneficial in promoting e-reporting adoption and improving the quality and quantity of reports in e-reporting. One barrier to e-reporting implementation is the lack of training and instructions. The request for education and training was also identified by a previous study, in which it was found that the reporters might not understand the importance of patient safety reporting or did not know how to report a case without sufficient education in the organization [17]. As for the lack of assistant designs in e-reporting, reporters may feel it difficult to report a case without certain helps from the system. It is consistent with the evidence that the under-reporting among health care professionals was due to not knowing the need or ability to report, not knowing what to report, or not knowing how to report [18-20]. Therefore, the training of patient safety reporting in the department would be of help in removing the barriers to e-reporting adoption. On the other hand, a user-centered design would improve user acceptance of such systems and greatly substitute the need for user training.

The confusion about taxonomies in e-reporting was another issue reported by the respondents. Taxonomies in e-reporting should function as a guide to what to report. However, reporters who are used to the everyday language of patient safety used during work, may not be familiar with the terminology of the classifications [21]. The perception of the taxonomies would influence the consistency and correctness of data if the items in the taxonomies are hard to understand and the understanding depends on reporters’ personal opinions. Additionally, the difficult and not-usable taxonomies in e-reporting would also increase the difficulty of use for reporters and discourage reporters’ future reporting. This may indicate a need for taxonomies that are more intuitive along with examples or other help documents to assist reporters’ understanding.

One crucial barrier to use of e-reporting is the lack of time among reporters. In other words, e-reporting might not be efficient enough for reporters considering their busy schedule. This echoed the findings from other studies in which the time and effort needed for reporting has been the primary self-reported barrier to reporting [22]. On one hand, physicians, nurses, as well as other healthcare professionals can be extremely busy and have no time to report. On the other hand, some of the reporting systems may require too much time and effort to report a case. From the perspective of health care professionals’ schedule, there should be a certain period of time arranged for these potential reporters’ to ensure they have the chance and energy to report the cases they have observed. As for the design of e-reporting, more thought should be put into solving the burden and complexity of the system to save time and effort for the reporters.

The other barrier to e-reporting adoption is the lack of feedback. As discovered in other studies, inadequate feedback has been recognized as one of the most significant barriers to reporting [23,24]. Although data from HSOPS has shown that most people have been given feedback about the patient safety condition in the hospitals, the information was limited to the error happened, changes put into place and ways to prevent future errors [8]. In fact, various aspects of feedback were expected but not received by most respondents in our study. The lack of feedback was thought to be a loss of control and a shortage of education as well. With respect to the control of the whole reporting process, a reporter may want to have a better understanding of the progress and his or her performance of the reporting behavior through the feedback provided by the system or the reviewers. For example, the reporter may want to know what percentage of the content has been completed, how much time is needed to finish the report, the completeness of the report, the correctness of the report, whether the report is successfully submitted, whether the reviewer has reviewed the report and so on. As for patient safety education and prevention, reporters may expect to receive feedback with lessons learned from the cases and information about what has happened and what will happen, such as the causes of the cases, the prevention methods, the response actions, and the policy change [25]. Therefore, comprehensive feedback may be helpful in promoting e-reporting usage by increasing user control and in promoting patient safety education and prevention.

### Feedback on the proposed user-centered features

In this study, four user-centered learning features and one group of assistant features were introduced and asked for feedback. These features were aimed to facilitate reporters’ adoption of e-reporting as both a quality reporting tool and a platform for learning. Users’ perceptions of the features were gained from the interviews to suggest the future direction of useful e-reporting designs.

The feedback on the proposed user-centered designs revealed a need for designs that could narrow the gap between the expectations of the reporters and the functionality of current e-reporting. The participants tended to link their needs with their preference of the proposed features in the interviews. As aforementioned, the reporters were expecting efficient and useful systems while facing the problems of having busy schedules, limited instructions, and insufficient feedback according to their previous experience with e-reporting. On the other hand, the proposed user-centered features were thought to be possibly helpful because they could reduce time and effort, give control during the reporting process, provide guidance and instructions, and provide feedback and educational information. There is a connection between the perceived advantages of the proposed features and participants’ perceived benefits and barriers, which may support the needs for user-centered design in e-reporting that are tailored to reporters’ requirements, such as our proposed learning and assistant features. The results from the interviews have also shed some light on the importance of taking users’ evaluation of the system into consideration while designing e-reporting to increase satisfaction among users as well as the usability of the system [26].

However, there seemed to be a discrepancy between the results of the screening questionnaire and the responses from the interviews. In the interviews, the participants with positive attitudes toward the features mentioned more about the difficulties of using e-reporting. Conversely, the results from the questionnaire showed that those participants with a positive attitude toward the proposed features had fewer barriers regarding certain use of e-reporting, such as rating a severity score. The difference may due to the different questions included in the questionnaire and in the interviews. Nevertheless, the controversy may also indicate that a better acceptance of user-centered designs is related to other factors besides benefits and barriers to using e-reporting. On one hand, a reporter’s role and specialty could have an impact on the reporter’s expectation of e-reporting designs. System designs across an entire specialty may not be useful to every individual’s practices [14]. For instance, physicians tend to report severe cases while nurses report more cases at different severity levels [27]. Nurses may encounter more difficulties in evaluating the severity since they are reporting events of various severity levels. Therefore, assistant features facilitating rating a severity score may be more useful to a nurse than a physician. From another view of point, the previous experience with e-reporting may also contribute to a reporter’s perception of the system features. Inexperienced reporters, junior staff, and physicians would face more difficulties in reporting compared with experienced reporters [16,28,29]. Therefore, a new reporter would need more help and assistance from the system while experienced reporters may think the assistant features are not as helpful. Taking these two aspects into account, there should be further researches on the role of reporters and reporters’ experience with e-reporting to improve future system designs that meet different reporters’ needs in patient safety reporting.

## Limitations

First, the number of samples in the study may not be adequate to support the generalization of the results or explore the influencing factors of e-reporting adoption exhaustively. However, the number of participants in the study could be reasonable on the basis of the prevailing under-reporting among healthcare professionals with most people having no or limited experience with e reporting. In addition, reporters may not be willing to recall their experience related to patient safety reporting and get involved in a sensitive topic as patient safety events.

Second, the interviews were conducted by a hybrid approach by phone and face-to-face. The option for having a face-to-face interview or a telephone interview was given to the participants to accommodate health care professionals’ busy and unexpected clinical task assignments. It was assumed that the hybrid approach did not introduce any significant differences.

Lastly, the interviews were adapted to each individual’s experience of e-reporting for an in-depth description based upon an interview script in general. In order to improve the quality of information collection from the participants, questions asked in the interview were tailored into interviewee’s experience as the study progressed. Another inevitable factor was the learning effect of the interviewer along the way of doing interviews. Nevertheless, only one interviewer was assigned in the study and some extent of consistency can be thus ensured.

## Conclusion

The perceived benefits and barriers identified in this qualitative study supported the fragmented findings from previous studies. The perceived benefits of e-reporting include the convenience in data entry and sharing as well as the usefulness in promoting patient safety. The perceived benefits the participants presented are of guidance in the future e-reporting design. First, the convenience of e-reporting data entry may rely on the balance between structured and unstructured data entry. Second, e-reporting should support timely communication between the reporter and other interested personnel to ensure the convenience in data sharing. Third, the system design should focus on the functions that effectively support the improvement of patient safety so that the reporters would have better acceptance of the system.

As for the perceived barriers of e-reporting, the participants have demonstrated a lack of instructions and trainings, lack of reporter-friendly classifications, lack of time and lack of feedback. These difficulties suggested by the participants imply the future directions in e-reporting design. From the perspective of system designs, more assistant features should be provided to solve the problem of not knowing what or how to report a case. Assistant features may also be helpful in guiding reporters when having problems understanding or implementing the various classifications in e-reporting. Time and effort could be saved if the system was made easier to operate. And lastly, more learning features are needed in providing feedback and educational information that facilitate patient safety management and intervention.

In addition to the above critical issues in e-reporting, four learning features and a group of assistant features of e-reporting were presented and could be helpful in patient safety reporting. The first feature was a side panel that automatically summarizes the report, while the reporter is entering the data in e-reporting. The second feature was a table showing cases similar to the one the reporter is reporting with both the description and the analysis of the similar cases accessible. The third feature was a table showing reporters’ previous reports, including the review status, analysis of the cases and action being taken to resolve and prevent the case. The fourth feature was statistics with graphic features, such as a bar chart showing the number of events of the same type being reported each month/year. The last was a group of assistant features, such as data extraction from institutional systems and dynamic content that only shows relevant information. These proposed user-centered features, though not tested for usefulness, were well-accepted by the participants in the interviews and thought to possibly help the reporters during their reporting process.

## Figures and Tables

**Table 1:** Select learning features for enhancing e-reporting system*

Feature	Description
**A side panel**	automatically summarizes the report while the reporter is entering the data in e-reporting
**A table**	showing cases similar to the one the reporter is reporting with both the description and analysis of the similar cases
**A table**	showing reporters’ previous reports, including review status, analysis of cases and action being taken to resolve the cases
**Graphical statistics**	such as a bar chart showing the number of events of the same type being reported each month/year

* an abridged version of the complete feature demonstration developed by the team based upon an incorporation of prevailing features, literature suggested functions and design concepts [11].

**Table 2:** Questionnaire items

**1**	Role of user
**2**	Intention of using e-reporting
**3**	Ease of use
**4**	Time needed and expected to report a case
**5**	The difficult and time-consuming contents to fill in a report
**6**	Security and anonymity
**7**	Benefits of using e-reporting
**8**	If participant considers reporting a case as his/her responsibility
**9**	Preference of alternative reporting method
**10**	Individual barriers
**11**	Resource and technology barriers
**12**	Organizational barriers
**13**	If participant is often delegated to report a case
**14**	If classification is easy to understand.
**15**	If participant is familiar with any classification
**16**	If participant has ever received feedback
**17**	Preference of the feedback methods
**18**	Importance of feedback
**19**	Preferred frequency of receiving feedback
**20**	Preference of the listed learning features
**21**	Preference of the listed assistant features
**22**	An open-ended question asking for comments and questions

**Table 3:** Topics discussed in the interviews

Major topics	Specific questions
**Experience with e-reporting**	Frequency of use of e-reporting; period of time of using e-reporting
**Benefits of e-reporting**	Advantages of e-reporting; advantage compared to alternative reporting methods; Improvement in patient safety after the use of e-reporting
**Barriers to e-reporting**	Difficulties of using e-reporting; time needed to use e-reporting; feedback of the patient safety events; other barriers
**Perceptions of the proposed learning features**	Usefulness of the feature
	Reasons for the usefulness/lack of usefulness of the feature

**Table 4:** Summary of perceived benefits and barriers

	Themes	Main findings
**Benefits**	Convenience in data entry and sharing	e-reporting has made the data entry and information sharing quick and simple.
		e-reporting was considered more convenient than the alternative ways of reporting patient safety events, such as paper-based systems, writing emails, and oral reporting.
	Improvement in patient safety	e-reporting was helpful in detecting problems, analyzing events and providing solutions.
		e-reporting may help bring actual improvement in patient safety which was not believed due to poorly designed systems.
**Barriers**	Lack of instructions and trainings	The lack of training in patient safety reporting has led to reporters’ unawareness of e-reporting.
		The lack of education on event analysis has made it hard for reporters to report the cases.
		The lack of instruction features in e-reporting has made the use of e-reporting difficult.
	Lack of reporter-friendly classification	The classifications of patient safety events were hard to understand and utilize in the real world.
	Lack of time	The lack of time for reporting has affected reporters’ usage of e-reporting.
	Lack of feedback	There was a lack of feedback in patient safety reporting.
		The feedback expected by the reporters varied, including feedback on the process of reporting, the analysis of the cases, and the intervention for future events.

**Table 5:** Summary of opinions on learning features

Features	Main perceptions
**A side panel that summarizes the report**	Increase control over the reporting process; provide guidance for data entry; assist accuracy check of the report
**A table showing similar reports**	Provide guidance for data entry and educational information about error prevention
**A table showing previous reports**	Provide feedback on the events
**Statistics with graphics**	Facilitate information comprehension
	Facilitate information sharing
